# Pubiotomy1Read before the Obstetrical and Gynecological Section of the Buffalo Academy of Medicine January 23, 1906.

**Published:** 1906-01

**Authors:** Max C. Breuer

**Affiliations:** Buffalo, N. Y.; Gynecologist to the German Hospital


### Pubiotomy.[1]

By MAX C. BREUER, M. D., Buffalo, N. Y.

Gynecologist to the German Hospital.

THE idea of severing the continuity of the pelvis temporarily, to facilitate delivery in narrow pelves, is an old one and originated very likely in France, where it had been a topic of academical discussion long before Jean Claude de la Curvée, a French physician practising in Warsaw, first performed symphysiotomy in 1644, after the death of the mother, for the purpose of saving the child. To the bold and ingenious French physician Sigault belongs the credit of having given to this idea life. He performed the operation on the 1st of October, 1777 in the Faubourg St. Denis in Paris and saved mother and child.

Sepsis, urethal and vesical injuries, discouraged obstetricians and brought the operation into disrepute, until Morisani, of Naples in 1866 took up the abandoned field, and encouraged by his results, Pinard in Paris, Zweifel in Leipsig, Schauta in Vienna and Dr. Harris of Philadelphia, performed symphysiotomy and published large numbers of cases, the best results being obtained by Zweifel, who saved in a series of 23 cases all the mothers and lost but two children. Such results could not be accomplished by Cesarean section, and one might wonder why this comparatively harmless and technically much easier operation than Cesarean section has not become more popular in this country. I believe the reason is threefold: first, the danger of tear of the bladder and urethra through the diverging ossa pubes; second, the hemorrhage from the tear of the corpora cavernosa of the clitoris; and, third, the reported loose joint and the resulting impairment of walk. A radical change of the matter in question has taken place, since Gigli, of Florence, hae executed the so-called pubiotomy or hebiotomy in 1894, although he himself admitted in the meeting of the Vienna Obstetrical Society, May 16, 1905, that to Champion belongs the credit of having first advocated and described even the technic of the operation, which, however, he never performed.

The indications for pubiotomy are in general the same as those for symphysiotomy,—that is, obstructed labor, where the delivery of a living child might be made possible by a moderate expansion of the pelvis. This condition will apply principally to simple flat, rachitic flat, and generally contracted pelves with a true conjugate of not less than 6.5 cm., a condition which would necessitate, if version or high forceps for good reason are not

1. Read before the Obstetrical and Gynecological Section of the Buffalo Academy of Medicine January 23, 1906.

to be considered, or have failed, Cesarean section or perforation of the living child. In examining a series of cases of pubiotomy at the Frauenklinik in Dresden, van Canoenberghe has found, that by a divergence of the severed pubic bone by 3 cm., the true conjugate increases 1 cm., the transverse diameter 1.4 cm., and each of the oblique by 1.3 cm. A divergence of 4 cm. does not lead to a tear of the capsular ligaments, such of 6 cm. might lead to a tear, but such injury would not be of serious consequences. Pubiotomy on both sides does not do more for the patient and retards recovery. The time to operate, of course, is not before the beginning of the second stage of labor; and, since in a number of cases of contracted pelvis, dilatation is often delayed by early rupture of membranes, artificial dilatation by Barnes's or Champetier de Ribes's bag or Bozzi's instrument has to be resorted to, before pubiotomy can be performed. If such artificial dilatation fails, Cesarean section or perforation of the living child has to be considered. When pubiotomy is done, the child ought to be delivered; the operation is preferably done on the side on which the occiput is going to pass through, or on the side of the feet of the fetus, if version is performed, and, if necessary, episiotomy on the side opposite to the pubiotomy should be performed. The hospital is the place to operate, and if infection has set in, one physician should perform pubiotomy, while another delivers the child.

Gigli himself has collected 85 cases out of the literature of the last years, the operations having been performed by Bar, Paris; Berry Hart, Edinburgh; de Boris, Rheims; Doederlein, Tübingen; Meiner, Amsterdam; Schauta, Vienna; van de Velde, Harlem; Walcher, Stuttgart; Zweifel, Leipzig, and others. In quick succession, cases are being reported in Germany, by Seligmann, Hamburg; Hohlweg, Kiel; Reiferscheid, Bonn; Dührssen, Leopold, and others.

During my recent work at the Gynecological Clinic of the University in Bonn, I saw two cases of pubiotomy, and since the operation is comparatively unknown in its details of the technic, I will describe those two cases as they were operated by Dr. Reiferscheid, the obstetrician in charge of the obstetrical clinic, and give the history of the cases.

Mrs. M. L., 31 years of age, primipara, with last menstruation on December 10, 1904. The pelvic measurments are: interspinal, 26 cm.; intercristal, 27 cm.; external conjugate, 17.5; diagonal, 8.5; true conjugate measured, according to Bylicki-Gauss, 6.75 cm. She entered the hospital on the 26th of September, at 10 A. M., two days after the beginning of labor and 29 hours after the rupture of the membranes.

Patient was sent in, to have Cesarean section performed. Status: fetus in I cephalic presentation; heart sounds distinct; os in fair dilation; head movable, has not entered the pelvis, sagittal suture in transverse pelvic diameter; labor weak, temperature normal; pulse 104.

At 7.30 P. M. dilatation is complete, labor pains are regular and strong, fetal heart sounds are normal, but no progress, temperature 99.

To deliver the child, left pubiotomy is performed by Dr. Reiferscheid during chloroform anesthesia. After the shaving of the pubes, an incision of 2 to 3 cm. is made immediately inside of the pubic spine, carried down to the horizontal ramus, and a curved trocar armed with a mandrin is carried back of the os pubis between bone and periosteum under control from the finger from the vagina, and brought out through a small incision outside of the left labium. After removal of the mandrin, the Gigli saw is led through the trocar, and after removal of the trocar, the bone is sawed through. Then the saw is removed (which, by the way, should not be done before the delivery of the child), and forceps is applied with no avail, since the pelvis does not dilate. The saw is reintroduced, and after one pull of the saw, which apparently cuts the ligaments in front of the pubic bone, the pelvis opens well, the separation of the two ends of the os pubis amounting to 5 or 6 cm. during forceps traction. A living male child is delivered without hemorrhage, after left episiotomy had been performed to avoid a perineal rupture.

Other operators recommend episiotomy on the opposite side. In examining the vagina, a rent 3 cm. long is found, beginning about 1 cm. below the urethra, which is deviated towards the right side. Catheterisation of the patient shows the urine to be free from blood. In the depth of the rent one palpates the separated pubes. The tear is closed with catgut. The upper incision is closed with silkworm-gut; the lower one drained; the episiotomy wound is closed with silkworm-gut, and adhesive Z. O. strips are laid around the pelvis. Twenty-four days afterward, the patient is allowed to get up and walks perfectly. There is good union; a callus can be felt distinctly in front, none back of the pubis.

Second Case: II para R. R., 36 years old. First child was born 4 years previously after a whole week's labor, by forceps delivery, and died right after delivery. Last gravidity undisturbed; last menstruation December 18, 1904. The pelvic measurements are: interspinal diameter 26.5; intercristal 27.5; external conjugate, 17.5; diagonal, 9.5; true conjugate, (according to Bylicki-Gauss), 7.25. Patient entered the hospital on the morning of the 27th of September. The membranes had ruptured two days previously without labor pains preceding; during the last 22 hours, good pains. Fetus in I cephalic presentation, fetal heart sounds varying between 120 and 170, os fairly open, its edges dilatable; sagittal suture in transverse diameter near the promontory; meconium passing.

In consideration of the danger to the child, pubiotomy is done in the same way as in the case before mentioned, only the saw is not removed before the delivery of an axphyxiated male child by forceps; no hemorrhage; wounds are dressed in the same way; Z. O. plaster strips accross the pelvis. Movements of pelvis after the third day are free from pain. Patient gets up on the 5th of October; walk is not changed, good union, good involution of genital organs.

From the observation of these two cases and from my study of the cases published during the last few years, I would say that pubiotomy can be performed safely by any gynecologist in proper surroundings, preferably in a hospital, in a very short time, and in emergency cases by any skillful practitioner who has some experience in surgery. The limit to the operation for mechanical reason is a true conjugate of above 6.5 cm.

The beginning of sepsis I do not consider a contraindication. I certainly would prefer pubiotomy to Cesarean section in a case, in which the temperature is rising. In an outspoken case of sepsis, I would very likely perforate the living child.

In my own observation, I remember having perforated a living child 16 years ago, as interne in the obstetrical clinic of the University of Breslau, on account of a subserous fibromyoma of the uterus, which obstructed the pelvis and made even craniotomy with following extraction a difficult operation.

During the last ten years of my practice here in Buffalo, I have never seen a case of such degree of contracted pelvis that version or high forceps, after waiting patiently for the configuration of the fetal head, could not be applied with good result; but I am accustomed to observe conscientiously the fetal heart sounds, and try at once to deliver the child, when I notice any signs of fetal weakness.

I have lost only two children by such proceedings; no mothers. But cases of higher degree of contracted pelvis are not as rare in Buffalo as I thought. An obstetrician with a consulting practice, told me that he had perforated six living children; another had perforated eight times. I have no doubt that some of these could have been saved by pubiotomy, and I venture to say, that mothers and relatives will consent to pubiotomy when they will refuse Cesarean section.

33 Allen Street.

